# Katz Fractal Dimension of Geoelectric Field during Severe Geomagnetic Storms

**DOI:** 10.3390/e23111531

**Published:** 2021-11-18

**Authors:** Agnieszka Gil, Vasile Glavan, Anna Wawrzaszek, Renata Modzelewska, Lukasz Tomasik

**Affiliations:** 1 Faculty of Exact and Natural Sciences, Institute of Mathematics, Siedlce University, Konarskiego 2, 08-110 Siedlce, Poland; vasile.glavan@uph.edu.pl (V.G.); renatam@uph.edu.pl (R.M.); 2 Space Research Centre, Polish Academy of Sciences, Bartycka Str. 18A, 00-716 Warsaw, Poland; sanna@cbk.waw.pl (A.W.); tomasik@cbk.waw.pl (L.T.)

**Keywords:** fractal dimension, time series, space weather, geomagnetic storms, geoelectric field

## Abstract

We are concerned with the time series resulting from the computed local horizontal geoelectric field, obtained with the aid of a 1-D layered Earth model based on local geomagnetic field measurements, for the full solar magnetic cycle of 1996–2019, covering the two consecutive solar activity cycles 23 and 24. To our best knowledge, for the first time, the roughness of severe geomagnetic storms is considered by using a monofractal time series analysis of the Earth electric field. We show that during severe geomagnetic storms the Katz fractal dimension of the geoelectric field grows rapidly.

## 1. Introduction

### 1.1. Motivation and Mathematical Basis

The pioneering work of B. Mandelbrot [1] was a starting point for a series of papers devoted to elaborate algorithms aiming to estimate the fractal dimension (fd) of nonlinear time series from records of measurements of various complex natural phenomena. The existence of a multifractal structure in the fluctuations of the heliospheric magnetic field’s strength, temperature and density was demonstrated in [2]. The paper by Potirakis et al. with an expressive title “Sudden drop of fractal dimension of electromagnetic emissions recorded prior to significant earthquake” [3] confirms once more the importance of the fractal dimension in studying local or global properties of the nonlinear time series associated with complex natural phenomena.

Our article is devoted to another natural phenomenon: the sudden increase of the fractal dimension in time series associated with the horizontal geoelectric field during geomagnetic storms. Therefore, we first give the necessary definitions of the Hausdorff measure and dimension of a set in a metric space with various algorithms to estimate this dimension, especially those connected with fractal time series. Among them, we mention the box-counting and Katz’s algorithm. We show here that during each severe geomagnetic storm the calculated Katz fractal dimension of geoelectric field, *E*, promptly rises, precisely at the time of increase of the *E* value. The geoelectric field fractal dimension based on the Katz approach demonstrates a change in the level of complexity of each severe geomagnetic storm.

### 1.2. Hausdorff Dimension

Consider the non-empty subset *F* in the Euclidean metric space $R^{n}$ and the real number δ>0. A countable family of subsets $U_{k}$ of diameter $diam(U_{k})\leq \delta$ such that $F\subset ⋃U_{k}$, is called a δ-cover. Given the real numbers s∈[0,∞) and δ>0, define

(1) $$H_{\delta }^{s}\left(F\right)=\inf\left\{\sum_{k=1}^{\infty }\;diam{\left(U_{k}\right)}^{s}\;:\left\{U_{k}\right\}{\;}_{k=1}^{\infty }\;is\;a\;\delta -cover\;of\;F\right\}.$$

As δ decreases, the class of δ-covers of the subset *F* is reduced, which in turn implies that the infimum increases. The quantity

(2) $$H^{s}\left(F\right)=\lim_{\delta \to 0}H_{\delta }^{s}\left(F\right)$$

is said to be the *s*-dimensional Hausdorff measure of *F* and the critical value of the parameter *s*, i.e., the value of *s* for which $\inf\left\{s\geq 0:H^{s}\left(F\right)=0\right\}=\sup\left\{s\geq 0:H^{s}\left(F\right)=\infty \right\}$ is called the Hausdorff dimension of *F* and is denoted by $\dim_{H}F$ (see [4,5]). As it is mentioned in [4], in some cases the covering may be made by balls of diameter at most δ, or by cubes of size at most δ. If one denotes the number of elements of such a covering by $N_{\delta }\left(F\right)$ and if, in addition, this measurement follows a power law, say $N_{\delta }\left(F\right)∼c\delta^{-s}$ for all s>0 and some constant c>0, then one obtains the following formula for the so called box-counting dimension:

(3) $$\dim_{B}F=\lim_{\delta \to 0}\frac{\log N_{\delta }\left(F\right)}{-\log\delta },$$

provided the limit exists. In general, $\dim_{B}F\leq \dim_{H}\left(F\right)$. Various box-counting-type formulae for fractal dimensions are used when studying self-similar fractals, as, e.g., the attractors of iterated function systems [6] with the middle third Cantor set, Sierpinski’s triangle or Sierpinski’s carpet, as particular cases, as well as when dealing with experimental data, such as time series, with some evidences of self-affinity.

### 1.3. Fractal Time Series

Given the stochastic process $X_{t}$ with t∈I as time, either discrete or continuous, let X(t),t∈I, be a path, or a time series, that realizes this process. Furthermore, let $r_{xx}\left(\tau \right):=E\left[x\left(t\right)x\left(t+\tau \right)\right]$ denote the autocorrelation function. The process $X_{t},t\in I,$ is said to possess short-range dependence, if $r_{xx}$ is integrable, i.e., if $\int_{0}^{\infty }r_{xx}\left(\tau \right)d\tau <\infty .$ Otherwise one says that the process has a long-range dependence.

A typical form of a nonintegrable autocorrelation function is when $r_{xx}\left(\tau \right)∼c\tau^{-\beta }$ as τ→∞, for some c>0 and 0<β<1. This is characteristic for so-called fractal time series: the heavy tail of the probabilistic mass function of X(t) makes $r_{xx}$ nonintegrable. Moreover, it is possible that the mean, as well as the variance of X(t), is not definite, so that for long-range dependent processes the concepts of mean and variance could be inappropriate to describe the local and global properties of time series. Instead of that, the fractal dimension and the Hurst parameter are used for this purpose.

As a measure of complexity of a time series, the fractal dimension illustrates how serrated or jagged its waveform is. The fractal dimension also discloses the scale of the randomness or of the unpredictability a process can exhibit. A fractal dimension close to 1.5 is an indicator of a Brownian time series. A fractal dimension between 1 and 1.5 indicates persistent behaviour: an increase in values will most likely be followed by an increase in the short term, and a decrease in values will most likely be followed by another decrease in the short term. At the same time, a fractal dimension between 1.5 and 2 is a sign of the antipersistent behaviour of the time series; an increase will most likely be followed by a decrease or vice versa. In addition to the above-mentioned, it is worth noting that while difference and differential equations are the main tools for studying classical stochastic processes such as moving average model (MA), autoregressive model (AR), autoregressive moving average (ARMA), autoregressive integrated moving average (ARIMA), etc. (see, e.g., [7,8]), fractal time series need a more sophisticated apparatus such as, e.g., fractional differential calculus (see, e.g., [9,10]).

**Figure 1:**
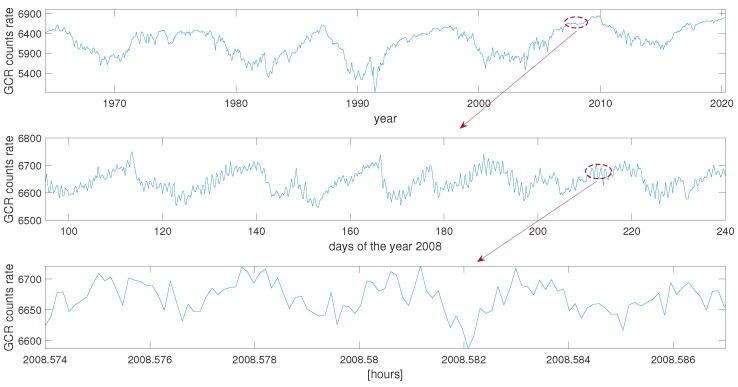
Temporal changes of the galactic cosmic ray (GCR) counts rate from Oulu neutron monitor: 27-day averages of daily data in the period April 1964–April 2020 (upper panel), half-day averages of 1-minute data in April 2008–August 2008 (middle panel), hourly data of GCR in 13–17 September 2008 (lower panel). Short periods marked by the brown dashed line in the upper panels are plotted with details in the lower panels displaying a self-similarity of the time series in various time-scales.

One of the applications of fractal theory is the examination of the changeability in solar activity [11], magnetospheric chaos [12,13,14,15] or cosmic rays [16]. This fractal character can be observed sometimes very clearly, as demonstrated by the galactic cosmic ray (GCR) measurements in Figure 1. The lower panel displays the 24-hour diurnal variation which is visible on the background of the 27-day GCR recurrence (middle panel), for which the background is the 11-year variability (upper panel).

Burlaga and coauthors [2,17] noted that the heliospheric magnetic field strength and components, treated as simple time series, have the features of fractal curves (see also [18]).

Multifractal properties of the geomagnetic storm were studied using various methods [19]. The generalized fractal dimension of the total magnetic field having peaks during the main phase of magnetic disturbances was used in [20] noting that fractal dimension changes demonstrate more details of solar activity in comparison to the Dst-index variations. Alberti et al. [21], using the multiscale generalized fractal dimensions in the SYM-H geomagnetic index analysis, showed a more steady behaviour at the timescales related to the solar wind changeability and to the nonlinear magnetospheric response to solar wind variations. An analysis based on the scatter-box-counting fractal dimension of the Dst-index exhibited fractal dimension reductions during magnetic storms [22], which was in an agreement with previous results [23], where a progressive complexity decreased during intense magnetic storms. As it was suggested in [12], based on power spectrum and Kolmogorov entropy analyses, the state of the magnetospheric response described by the AE-index was chaotic during the storm of January 1983. This result was confirmed recently by [24], when four chaos quantifiers and the multifractality approach were used in the analysis of the Dst-index data over the four solar cycles 20–23.

Here, we study the complexity of severe geomagnetic storms using a monofractal time series analysis of the geoelectric field *E*; to our best knowledge, this approach has never been used before.

Our aim in this article is twofold: (1) to show that the Katz fractal dimension is an adequate indicator of changes in the level of internal complexity of the geomagnetic storm articulated in the means of the local horizontal geoelectric field *E* ($E=\sqrt{E_{X}^{2}+E_{Y}^{2}}$, where the E-W direction is denoted *Y* and the N-S direction *X*); (2) to underline that the effects of geomagnetic storms might also be harmful to electrical systems of mid-latitude countries. For that purpose, we use a computed local geoelectric field, since it is the best proxy for geomagnetically induced current (GIC). This is particularly important in light of what has been shown in the matter of space weather events influence on the functioning of electrical power networks for mid-latitude countries in Europe: the Czech Republic [25,26], Poland [27,28,29], Italy [30,31], Greece [32], Spain [33] or Austria [34].

## 2. Data and Methods

### 2.1. Data

Solar activity varies from minima to minima, with maxima in between [35]. This activity affects all objects in the solar system. The links between solar activity and geomagnetic changeability are well known [36].

The region of near-Earth space, where the dominant role is played by the Earth’s magnetic field, is called the magnetosphere. Its shape and properties are formed by the interaction with the solar wind—the pressure of the solar wind compresses the field on the dayside of the Earth and stretches it into a long tail on the nightside [37]. The variations of the solar wind can cause a temporary disturbance of the magnetosphere. Such a phenomenon is called a geomagnetic storm [38]. The strongest geomagnetic storms are associated with the solar coronal mass ejection (CME), where a large amount of plasma with an embedded magnetic field interacts with the magnetosphere [39]. A similar response of the magnetosphere is caused by corotating interaction regions with high-speed solar wind streams originating in coronal holes [40]. Geomagnetic storms can last up to several days and can strongly impact technical infrastructure [41,42,43,44]. During a geomagnetic storm, the high energetic particles can penetrate the near-Earth space, causing satellites malfunction, and even increase the radiation risk to flight crews and passengers. Severe storms can also be responsible for damages to power lines and pipelines. Geomagnetically induced currents can cause transformer overheating and increase material ageing [32,45].

To estimate a semiquantitative level of geomagnetic activity, various geomagnetic indices have been introduced. Those indices are based on ground magnetic observations. The most frequently used indices are Kp, AE and Dst [46]. The geomagnetic indices are generally calculated from ground observatories and have their particular purpose. The Kp-index is designed to monitor global geomagnetic activity in a quasi-logarithmic scale, from 0 to 9. The procedure of computation is quite complex. The Kp-index is the arithmetic mean of the 3-hour standardized local K-indices of 11 northern and 2 southern stations between 44° and 60° north and south, respectively, geomagnetic latitude. Each of the K-indices is computed from the most distributed horizontal field components. The Kp-index is updated twice a month by the GFZ in Potsdam [47]. The Kp-index is expressed in a scale of thirds marked as: “o”, “+”, “−” and has 28 values, where “o”, “+”, “−” are represented by the values: 0, 1/3, −1/3, respectively. This index serves as a base for one of the NOAA Space Weather Scale of Geomagnetic Storms [48]. The scale provides information related to the storm frequency, its impact on the Earth environment and technical infrastructure. The scale starts with quiet conditions G0, through G1-Minor geomagnetic storms, with Kp = 5, G2-Moderate, with Kp = 6, G3-Strong, with Kp = 7, G4-Severe, with Kp = 8, including a 9− and finally, G5-Extreme, with Kp = 9. The severe geomagnetic storms, G4, which are the subject of our study in this article, can cause, e.g., surface charging and tracking problems in the case of spacecraft (even orbit correction may be needed) or low-frequency radio navigation disruptions. In power systems, extensive voltage problems may appear and protection system might be mistakenly activated. At that time, aurora can be visible even at low latitudes, such as 45°.

During the famous geomagnetic storm of 13 March 1989, the minimum value of the Dst-index was recorded being −589 nT [49] and the maximum value of 9 of the Kp-index was reached and kept for many hours. Tsurutani et al. (2003) [50] investigated a unique magnetic recordings from Bombay for the most powerful registered space weather event, i.e., the Carrington storm on 1–2 September 1859, estimating a Dst ≈ −1760 nT.

Here we consider first the Kp-index 3-hour values during the full solar magnetic cycle of 1996–2019, covering two solar cycles (SC), 23 and 24, extracting from this time series with more than seventy thousand data points the days when the maximal Kp-index was from the set 8, 8+, 9−. We obtained 31 days fulfilling this condition. In the literature one can find various categorizations of the storms, as well as substorms, e.g., [51,52,53].

**Figure 2:**
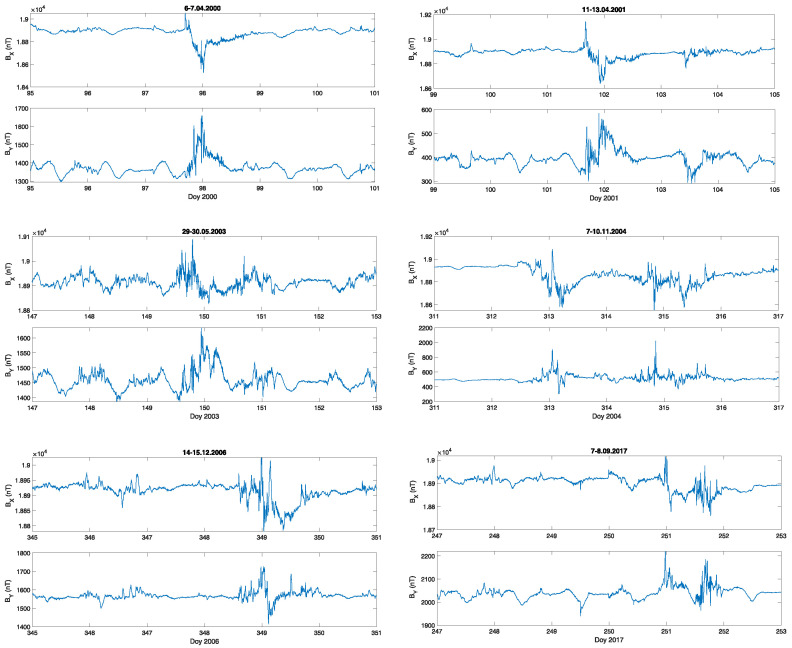
Geomagnetic field components, $B_{X},B_{Y}$, during the selected severe geomagnetic storms; the x-axis represents the day of year (Doy).

In the next step, the local geomagnetic situation was described by changes of one-minute data of two components of the geomagnetic field $B_{X},B_{Y}$ measured in Belsk, the Polish INTERMAGNET observatory. These components were used to determine the unique time series, that is, the local geoelectric field with a one-minute time resolution. For a detailed consideration, as examples, we have chosen six severe geomagnetic storms that lasted at least two days. The initial data of $B_{X},B_{Y}$ for these storms are presented in Figure 2.

### 2.2. Geoelectric Field

In order to compute the geoelectric field *E* from the geomagnetic data *B* we applied a 1-D layered Earth model, [54,55], in the frame of which the conductivity changes with the depth. More precisely, we considered *N* horizontal layers, each characterized by their own conductivity $\sigma_{n}$ and thickness $l_{n}$ (n=1,…,N). Then, the layered transfer function *K* (in the frequency domain *f*) can be described by the following recursive formula [54,56]:

(4) $$K_{n}=\eta_{n}\frac{K_{n+1}\left(1+e^{-2k_{n}l_{n}}\right)+\eta_{n}\left(1-e^{-2k_{n}l_{n}}\right)}{K_{n+1}\left(1-e^{-2k_{n}l_{n}}\right)+\eta_{n}\left(1+e^{-2k_{n}l_{n}}\right)},$$

where $K_{n}$ denotes the ratio of *E* to *B* at the top surface of layer *n* from $K_{n+1}$ at the top surface of the underlying layer n+1, $\eta_{n}=\frac{i2\pi f}{k_{n}}$, $k_{n}=\sqrt{i2\pi f\mu_{0}\sigma_{n}}$ and $\mu_{0}=4\pi 10^{-7}$ Hm${}^{-1}$ [55]. The initial value in Equation (4) corresponds to the case when the layer n=N is a uniform half-space and $K_{N}=\sqrt{\frac{i2\pi f}{\mu_{0}\sigma_{N}}}$. The final value $K_{1}$ (n=1) is the transfer function relating *E* and *B* at the Earth’s surface [55,57].

It is worth underlining that real observations of the geoelectric field are unfortunately rare, thus we are dependent on the data obtained as a result of mathematical modelling, e.g., [58].

Here, to perform the analysis of 1-minute geomagnetic field data from the Belsk station, we applied the Earth model number 45 [59], which is dedicated to the Poland region, and is used in a number of studies [27,60,61]. In model 45, we have four layers with, from the top down, the following thicknesses and resistivities (1/σ): 6 km, 5 Ω· m; 105 km, 1000 Ω· m; 300 km, 100 Ω· m; above a half-space of 10 Ω· m. In the next step of the analysis, we decomposed the geomagnetic field $\left\{B_{X},B_{Y}\right\}$ into its frequency components $\left\{B_{X}\left(f\right),B_{Y}\left(f\right)\right\}$ and next multiplied by the corresponding transfer function values, namely $E_{X}\left(f\right)=K\left(f\right)B_{Y}\left(f\right)$ and $E_{Y}\left(f\right)=-K\left(f\right)B_{X}\left(f\right)$, where $E_{X}\left(f\right)$ and $E_{Y}\left(f\right)$ denote the geoelectric field frequency components. Finally, we employed the inverse Fourier transform to obtain the value of a geoelectric field in the time domain E(t) for both northward ($E_{X}$) and eastward ($E_{Y}$) components [54,62]. It is worth noting, that this 1-D model is fast and accurate at a single location [58], which is the case of our studies.

### 2.3. Katz Fractal Dimension

In the literature, one can find various methods of fractal dimension estimation (e.g., [63,64,65]). Here we apply the Katz fractal dimension method [66], in the frame of which the roughness of the two-dimensional profile of a univariate time series is considered.

Let us consider a time series of real measurements *(t, X(t))*. For the particular subset *F* the mean *M* and the sum *L* of the Euclidean distances between the successive points of the subset *F*, as well as the diameter *d* of the subset *F*, which in this case coincides with the maximum distance between the first point and any other point of *F*, are calculated. Katz’s formula [66] for the fractal dimension of the subset *F*, considered as a waveform, looks as follows:

(5) $$fd_{K}=\frac{\log(w)}{\log\left(w\right)+\log\left(\frac{d}{L}\right)},$$

where $w=\mathrm{int}(\frac{L}{M}$) is the number of steps in the considered waveform describing the time series *(t, X(t))* (int(∗) denotes the integer part of *). Here, the normalisation $w=\frac{L}{M}$ was used to avoid an issue linked to the selection of the greatest distance, which would lead to the fractal dimension being dependent on the units used in the measurements.

Katz’s estimator for the fractal dimension has been used in mechanical issues, such as a crack identification in beam structures [67], as well as in signal pattern recognition [68] or heart rate variability analysis [69]. In intracranial electroencephalogram data studies it was shown [70] that the Katz approach revealed discriminating power.

Here, we took into account 1-minute data of computed geoelectric field *E* during the whole week around a particular storm; thus, we had for each case a time series consisting of 10,080 *E* data points. Time series of the computed geoelectric field presented rather lognormal distributions.

In the next step, for each consecutive 60 min of the time series, we have computed the Katz fractal dimension. Thus, for each storm we obtained temporal changes of the fractal dimension with 1-hour resolution during the whole week.

Since in the literature there appear disclosures about underestimating the fractal dimension using Katz’s method, e.g., [71], we consider also the estimation formula for the fractal dimension for a planar curve, proposed by B. Mandelbrot [1]:

(6) $$fd_{2}=\frac{\log(L)}{\log(d)}.$$

Elementary manipulations show that $fd_{2}<fd_{K}$, at least for positive log*M*. In the case of logM<0 the inequality reverses, which agrees with the claim of C. Sevcik that in case of a high sample frequency, i.e., a large number of steps, the Katz formula underestimates the Hausdorff dimension of the waveform [71,72].

## 3. Results and Discussion

### 3.1. Katz Fractal Dimension of Geoelectric Field

**Figure 3:**
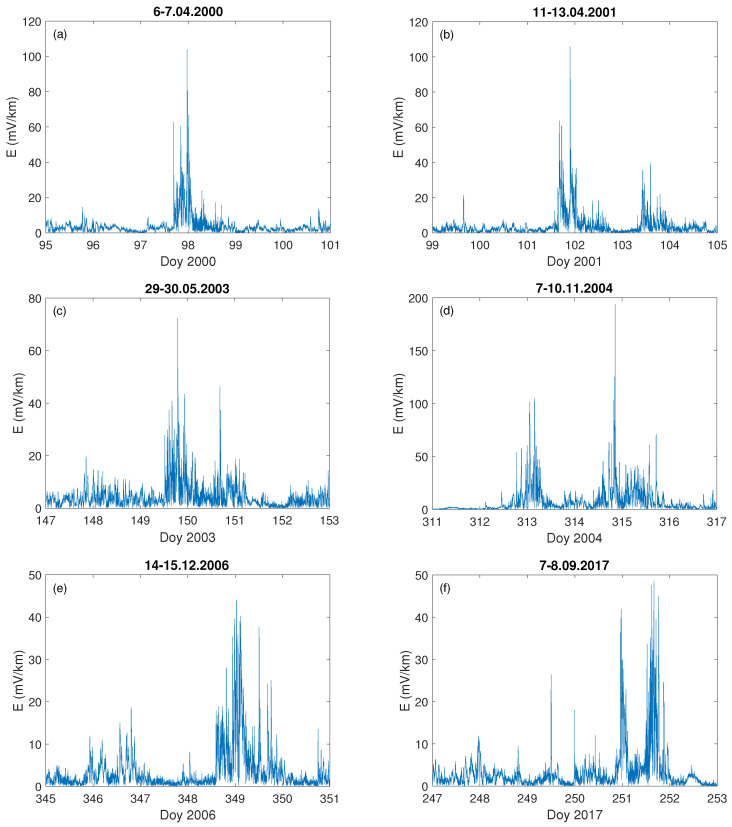
Geoelectric field, *E*, during the selected severe geomagnetic storms: (**a**) 6–7.04.2000, (**b**) 11–13.04.2001, (**c**) 29–30.05.2003, (**d**) 7–10.11.2004, (**e**) 14–15.12.2006 and (**f**) 7–8.09.2017, on the x–axis is marked the day of year (Doy).

The basic time series subjected in our study to the fractal dimension analysis is the local horizontal geoelectric field *E*, computed with the aid of the 1-D layered Earth model. Since there are no geoelectric field measurements in Poland, and, moreover, this kind of measurement is rare, the need of modeling arises [58]. We have verified our computed *E* against the available real measurements form Japan [73] and UK [58] and there is a reasonable agreement between the measured and computed *E* values. Figure 3 presents six examples from the 31 analysed cases of the calculated geoelectric field, *E*, during the severe geomagnetic storms that took place during the last two solar cycles, 23 and 24.

**Figure 4:**
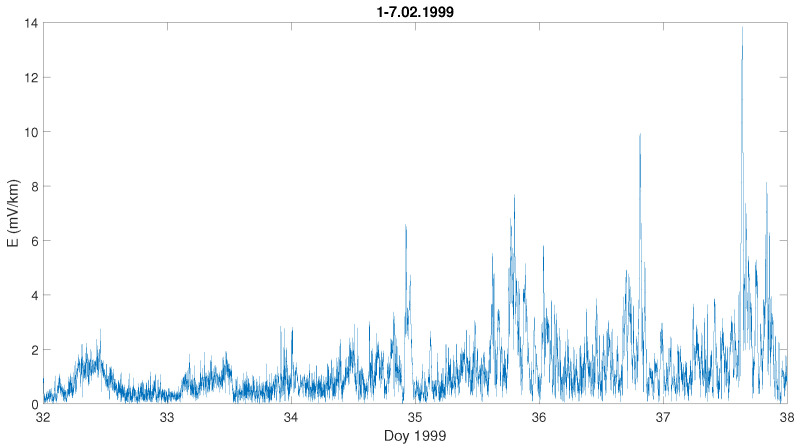
Temporal changes of the geoelectric field during quiet days at the beginning of February 1999; the x-axis indicates the day of year (Doy).

**Figure 5:**
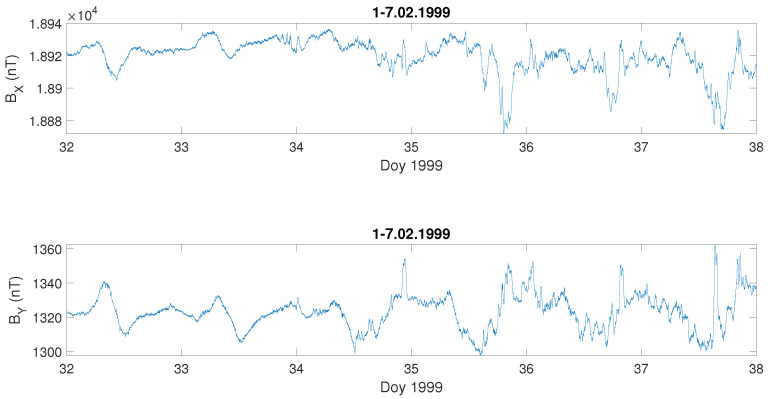
Temporal changes of the geomagnetic field components, $B_{X},B_{Y}$, during quiet days at the beginning of February 1999; the x-axis indicates the day of year (Doy).

We can observe that during the severe storm (data of the storm are given on the top of each Figure 3a–f), the local geoelectric field grows rapidly, reaching even 200 mV · km${}^{-1}$, as it was during the storm on 7–10 November 2004 (Figure 3d), while in Figure 3e) it is the least intense (in the sense of the geoelectric response) during the geomagnetic storm of 14–15 December 2006. For comparison, we present also a temporal behaviour of the Earth’s electric and magnetic field under undisturbed geomagnetic conditions (Figure 4 and Figure 5). One can see in Figure 4 that during a quiet time, the value of the electric field is just around 12 mV · km${}^{-1}$, much lower than during the storm occurrence.

**Figure 6:**
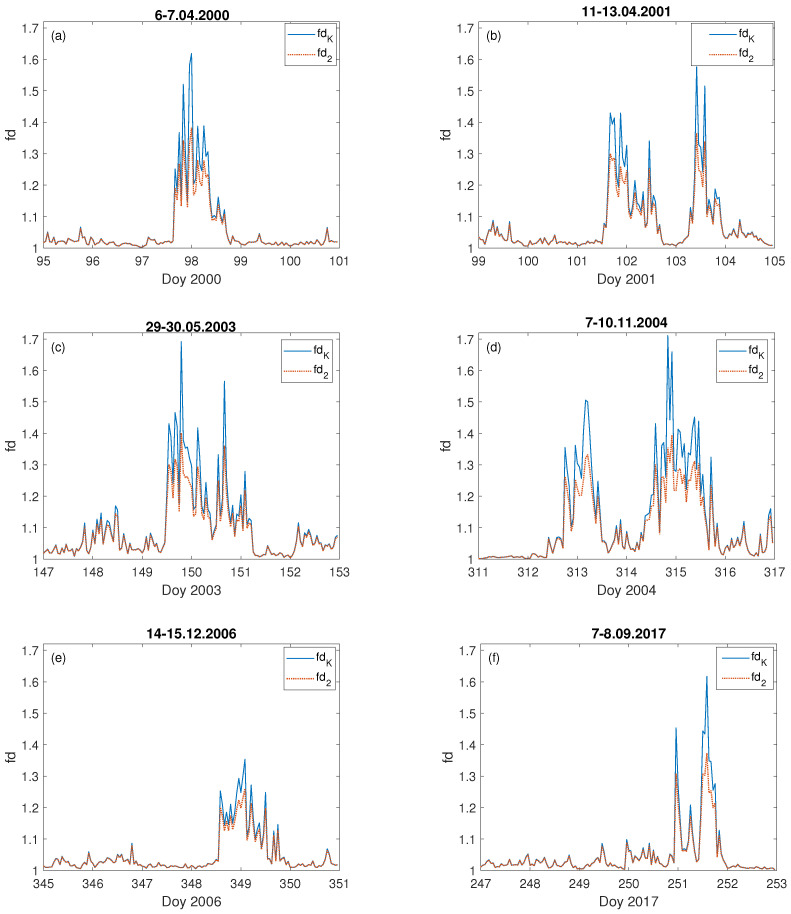
Fractal dimension: $fd_{K}$ (blue straight line) and $fd_{2}$ (red dashed line) of geoelectric field during the selected severe geomagnetic storms: (**a**) 6–7.04.2000, (**b**) 11–13.04.2001, (**c**) 29–30.05.2003, (**d**) 7–10.11.2004, (**e**) 14–15.12.2006 and (**f**) 7–8.09.2017, on the x–axis is marked the day of year (Doy).

Figure 6 presents the fractal dimension values, $fd_{K}$ (Equation (5)) and $fd_{2}$ (Equation (6)), calculated for six examples (the same storms as for the computed *E*, see Figure 3) from all the studied severe geomagnetic storms which took place during the last two solar cycles, 23 and 24. For all the analysed cases the increase of the fractal dimension much above 1 occurred exactly at the time when *E* was rising. One can see, that the temporal behaviour of $fd_{K}$ and $fd_{2}$ has precisely the same character, although with values sometimes greater for $fd_{K}$ than $fd_{2}$.

**Figure 7:**
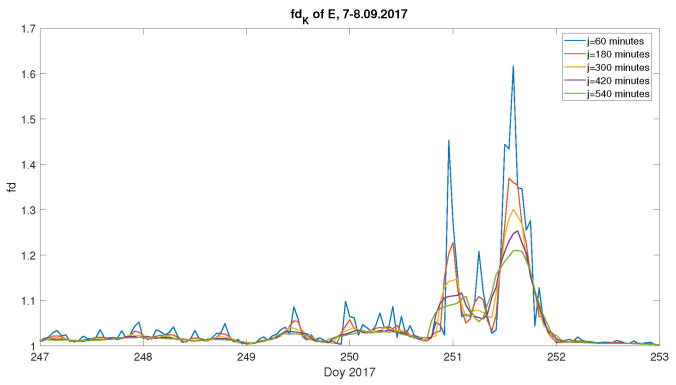
Fractal dimension computed with the Katz approach, with moving windows of various lengths j = 60, 180, 300, 420 and 540 min, for the September 2017 Storm; the x-axis indicates the day of year (Doy).

**Figure 8:**
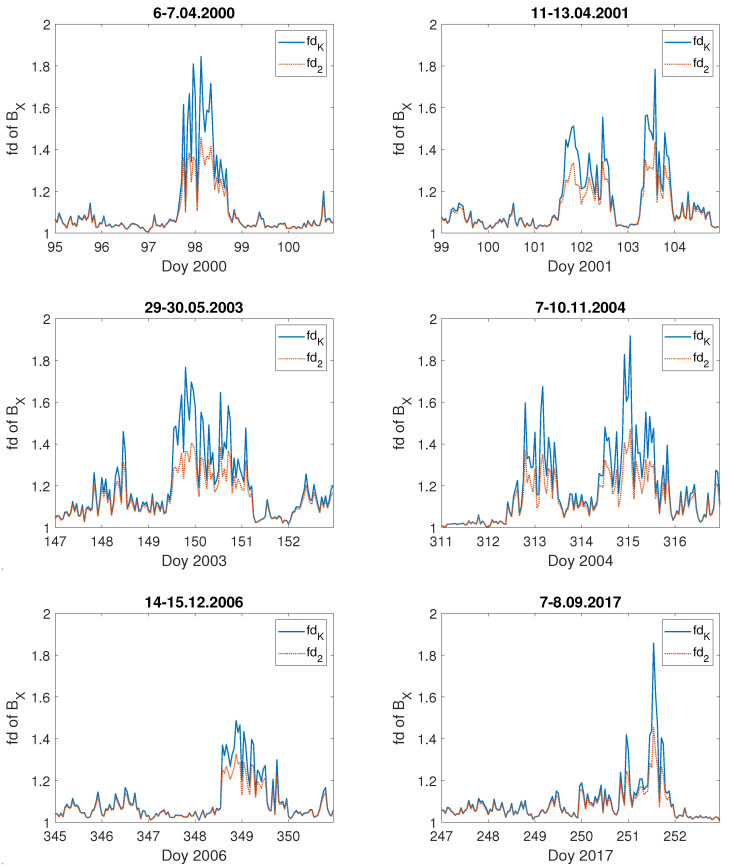
Fractal dimension: $fd_{K}$ (blue straight line) and $fd_{2}$ (red dashed line) of geomagnetic $B_{X}$ field component during the selected severe geomagnetic storms; the x-axis indicates the day of year (Doy).

It is worth noting, that the maximal value of the fractal dimension of *E* is not always associated with the largest growth in the geoelectric field value. For instance, values of *E* during the storms of 14–15 December 2006 (Figure 3e) and 7–8 September 2017 (Figure 3f) are similar, being not greater than 50 mV · km${}^{-1}$. However, the fractal dimension of *E* during the first mentioned storm was less than 1.4 (Figure 6e), while for the second one it was above 1.6 (Figure 6f), which is comparable to the fractal dimension characterising the 7–10 November 2004 (Figure 6d) storm when the value of the geoelectrical field (Figure 3d) was the highest among the presented examples. However, for the geomagnetic storm of 14–15 December 2006, *E* values are the lowest (below 45 mV · km${}^{-1}$) with the lowest fd values (below 1.4), as well. It was also the case for the quiet time period (Figure 4 and Figure 5). The fractal dimension changeability suggests substantial differences in the level of storm complexity. Comparable results were obtained recently by [24,74], showing that during major geomagnetic storms the strongest nonlinearity features occur. Moreover, there is no unequivocal dependence of the fractal dimension extremes on the maximum value of the Kp-index, as most of the storms shown in Figure 6a–f, were characterized by the maximum value of Kp = 8+. Only the storm presented in Figure 6d had a maximum value of Kp = 8. This demonstrates that during the storm the fluctuation’s structure changes and the Katz fractal dimension may, somehow, describe its internal properties.

We are aware that usually a few hundred data points are used for the fractal dimension estimation and computations using only 60 data points might be treated as insufficient. For that reason, we have performed computations with moving windows of various lengths. Figure 7 presents the Katz fractal dimension values for the computed geoelectric field during the September 2017 geomagnetic storm, with moving windows of the following lengths: j=60, 180, 300, 420 and 540 min. Figure 7 reveals that the overall behaviour is kept for all the windows lengths with a clear distinction of the geomagnetic storm appearance. Moreover, the results with j=60 min reveal much more details (which is natural) than for higher *j*. It has to be underlined that with longer windows, some local features will be lost, but the fractal dimension better describes the signal with a statistical significance, over broader scales. Although, the fractal dimension in time series meets some kind of self-affinity of the waveform, which in turn implies the analysis of large enough windows, the estimation based on relatively short ones discloses the local properties of time series. The latter corresponds to the main goal in our studies. Therefore, these analyses suggest that j=60 min is a reasonable compromise, if one intends to study very local effects.

**Figure 9:**
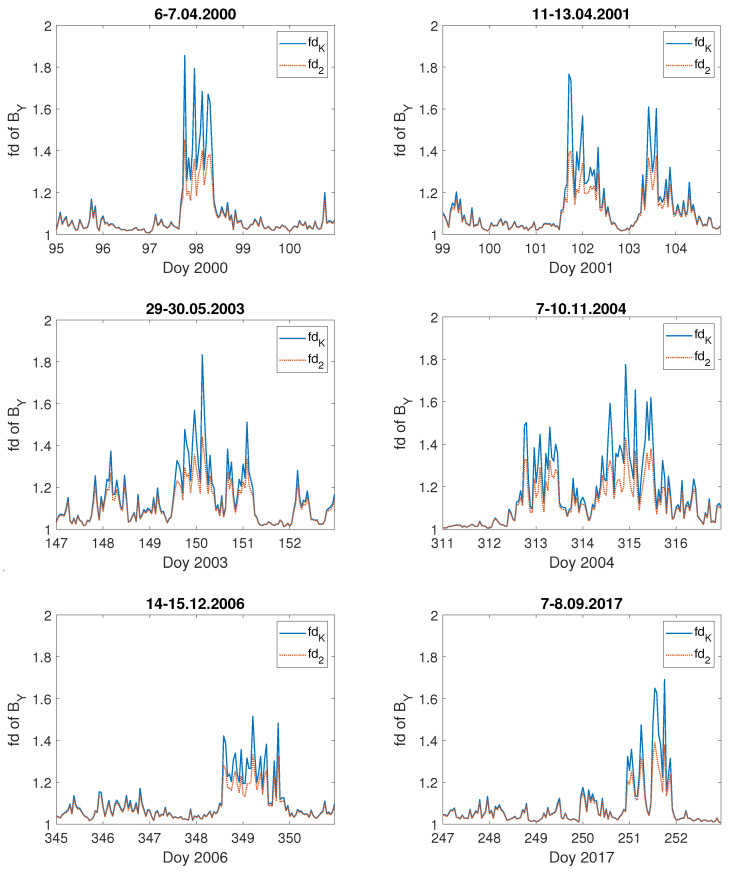
Fractal dimension: $fd_{K}$ (blue straight line) and $fd_{2}$ (red dashed line) of geomagnetic $B_{Y}$ field component during the selected severe geomagnetic storms; the x-axis indicates the day of year (Doy).

For comparison, we present the fractal dimension computed for the geomagnetic field components $B_{X},B_{Y}$ for these storms—Figure 8 and Figure 9, respectively. One can see that during these severe geomagnetic storms the fractal dimension of the components $B_{X},B_{Y}$ of the geomagnetic field increases simultaneously with the extreme changes in the values of $B_{X},B_{Y}$. It is worth noting that the fractal dimension of the primary $B_{X},B_{Y}$ geomagnetic component data is a bit higher than for the geoelectric field *E*. Thus, since space weather also affects via GICs the functionality of the electrical power networks at mid-latitudes [25,27,30,32,33,34], it is worth studying the properties of the local geoelectric field, as it is treated as the best proxy for the geomagnetically induced currents.

**Figure 10:**
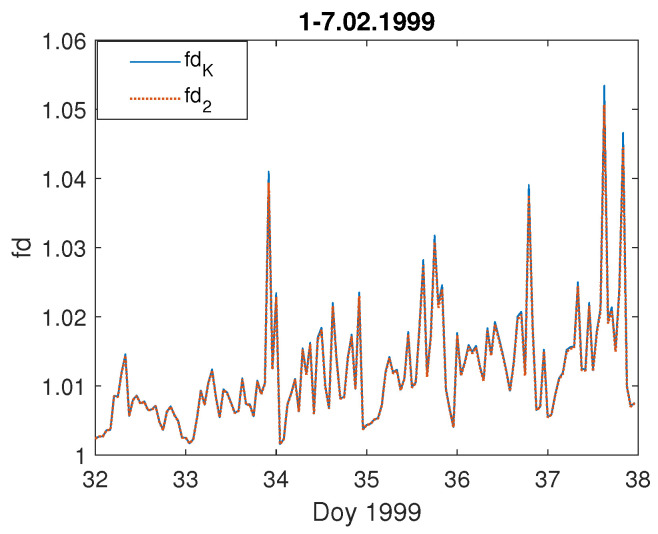
Temporal changes of the fractal dimension: $fd_{K}$ (blue straight line) and $fd_{2}$ (red dashed line) of the geoelectric field during quiet days at the beginning of February 1999; the x-axis indicates the day of year (Doy).

**Figure 11:**
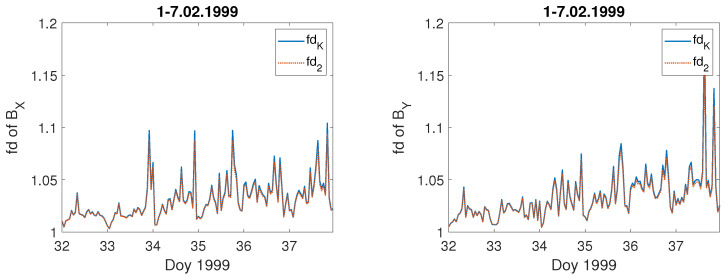
Temporal changes of the fractal dimension: $fd_{K}$ (blue straight line) and $fd_{2}$ (red dashed line) of the geomagnetic field components, $B_{X}$ (left panel), and $B_{Y}$ (right panel) during quiet days at the beginning of February 1999; the x-axis indicates the day of year (Doy).

Figure 10 shows that, when the geomagnetic conditions are peaceful, the fractal dimension of the electric field is around one, much lower in comparison with that during the storm appearance. Moreover, the fractal dimension values obtained using both methods (Equations (5) and (6)), $fd_{K}$ and $fd_{2}$, for a quiet interval are exactly the same, i.e., blue straight line and red dashed line cover. It is also the case for the geomagnetic field components $B_{X},B_{Y}$ fractal dimension computed for the same quiet period (Figure 11).

As already mentioned above, various geomagnetic indices are used in the literature to describe the nature of individual storms, such as the Dst-index with the southwardly directed Bz component of the heliospheric magnetic field [53], the SYM-H [75], the AE-index [76], PC [77], and many others. The multitude and variety of these parameters make the unambiguous characteristics of storms extremely complex. However, for us, it is also a determinant of the direction of further research aimed at the best possible characterization of the complexity of individual storms.

### 3.2. Usefulness and Limitations of the Katz Fractal Dimension

In various medical studies, the usefulness of the Katz approach was shown [70,77]. This fractal dimension seems to emphasize the actual meaning of the considered fd type as a characteristic of the roughness of the two-dimensional profile of a univariate time series. Hence, the link between fractal dimension and anti-/persistence (generally expressed by the Hurst exponent *H*) can be justified. The fractal dimensions for persistent, antipersistent and Brownian dynamics then satisfy the expression fd+H=2, which is accurate for Gaussian processes, but generally wrong for heavy-tailed processes.

In many articles it was shown that the Katz fd is underestimating the fractal dimension of synthetic, i.e., ideal data, and is sensitive to a change of the amplitude [78]. Bearing this in mind for a fuller consideration of the fractal nature of data, we have applied two additional approaches to fd estimation, namely, those proposed by T. Higuchi [79] and C. Sevcik [80].

In Sevcik’s fd approach [72] the following normalisation was proposed: $t_{i}^{*}=t_{i}/t_{max}$ and $x_{i}^{*}=\left(x_{i}-x_{min}\right)/\left(x_{max}-x_{min}\right)$, where $t_{max}$ and $x_{max}$ are the maximal values of $t_{i}$ and $x_{i}$, respectively, and $x_{min}$ is the minimal value of $x_{i}$. The following approximation of the box-counting dimension was used [72]: $fd_{S}=1+\frac{\log2L}{\log2(N-1)}$. For this approach it was shown in tests on the ideal, synthetic data that there were situations when it overestimated, as well as underestimated, the theoretical fractal dimension [78].

Higuchi’s approach [79] is based on the construction of *k* (interval time) subseries, with the initial time *m*: $x_{km}:x_{m},x_{m+k},x_{m+2k},\ldots ,x_{m+\mathrm{int}((N-m)/k)k}$, for m=1,…,k. For each subseries, its length estimator looks as follows: $L_{m}\left(k\right)=\frac{1}{k}[(\sum_{i=1}^{\mathrm{int}((N-m)/k)}|x_{m+ik}-x_{m+(i-1)k}\left|)\right]\frac{N-1}{\mathrm{int}((N-m)/k)k}$. It has to be underlined that the latter is not a length in Euclidean sense, as it was in the Katz’s formula Equation (5). It is the normalized sum of the absolute values of the differences in ordinates of couples of points, taken at a time interval equal to *k*. L(k) is calculated as the average of *k* values of $L_{m}\left(k\right)$. This average length is proportional to $k^{-fd_{H}}$, and the slope $fd_{H}$ defines the Higuchi fractal dimension value. One can see that the Higuchi fractal dimension allows the study of the behaviour of a characteristic quantity over different scales (similarly to Hausdorff’s original concept, as well as classical box-counting or generalized Rényi dimensions). On the other hand, in contrast to Katz or Sevcik’s approach, the Higuchi algorithm requires to assume the $k_{max}$ (the maximal number of subseries) parameter value, which may disturb the fractal dimension we are looking for.

Hence, these algorithms differ in terms of accuracy, sensitivity to the sampling frequency and dependency of the estimation on the selected length of the time window. We performed numerous tests of various fractal dimension approaches to verify our methodology. Following Raghavendra and Dutt [78], we generated synthetic waveforms: Weierstrass, Weierstrass–Mandelbrot, Takagi and Fractional Brownian motion. This analysis showed that the best at reconstructing the original fractal dimension of ideal, synthetic data was Higuchi’s method. As for Sevcik’s approach, there were situations when it overestimated, as well as underestimated, the theoretical fractal dimension. It also demonstrated that for synthetic data, Katz’s method was underestimating the fractal dimension of ideal waveform, at least for large enough windows.

**Figure 12:**
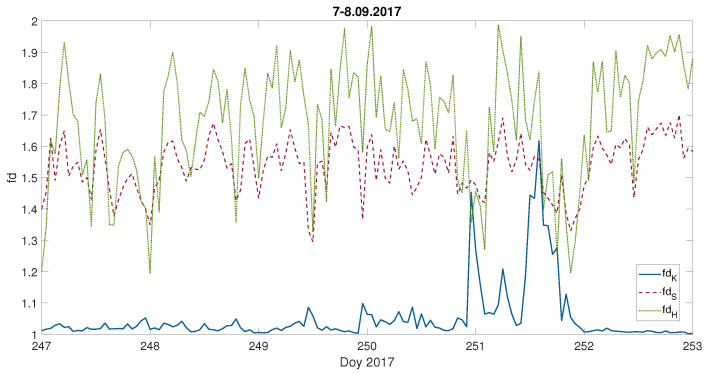
Fractal dimension computed with the Katz $fd_{K}$, Sevcik $fd_{S}$ and Higuchi $fd_{H}$ (with $k_{max}=12$) approach, for each 60 min, during the September 2017 geomagnetic storm; the x-axis indicates the day of year (Doy).

However, one has to always bear in mind that we are not working with ideal data, and the nature of real data is very complicated. It could be the case that we are dealing with data for which a more appropriate description is presented in [70] (Figure 2), where the Katz fd works much better than Higuchi’s fd. Having all this in mind, we conducted a series of tests for the recent September 2017 geomagnetic storm. An example of our tests is represented in Figure 12. It shows that the Higuchi and Sevcik fds behave similarly, being almost parallel, with values differing from each other and from the Katz fd values. Moreover, the Higuchi and Sevcik fds manifest a diurnal wave, which is planned to be studied more deeply in future work. Finally, Figure 12 displays that the Katz fd clearly indicates the geomagnetic storm appearance. Although, there is some tendency in the Higuchi and Sevcik fds to suffer a drop around the storm period. It might be compatible with [81] results, based on the one-minute data of the SYM-H index, showing stronger dynamical irregularities appearance during the quiet than during the storm time intervals. This result was in an agreement with the previous [82] distinction between quiet and disturbed time intervals, made on the base of the Dst-index investigation.

We are aware that for quiet times the Katz fractal dimension strongly underestimates the fractal dimension of the time series. However, Figure 12 shows that during the storm all the fractal dimensions have an inclination towards similar values, so it might be that during the storm period the real fractal dimension is close to the value given by the Katz fd. At the same time, the Katz fractal dimension seems to be much less sensitive to small changes, in contrast with the Higuchi method, which can show up strong instability under small perturbations of the time series by a small constant ϵ (0<ϵ<<1), as it was shown by Liehr and Massopust [83]. The advantage of Katz over Higuchi’s approach consists of the fact that a $k_{max}$ parameter [84] is superfluous. Yet, it has the downside of requiring a longer time series to achieve significant results. This led us to the judgement that the Katz algorithm is more efficient in detecting sharp/rapid signal changes typical of geomagnetic storms than the smaller, slower, and more gradual changes in quiet periods. Hence, the strong amplitude response of the Katz fd might be its advantage, as in our case, as well as a disadvantage.

## 4. Summary

We have computed the local geoelectric field, *E*, based on the Belsk magnetometer data, with one-minute resolution during the full solar magnetic cycle of 1996–2019, covering two solar activity cycles, 23 and 24. It served us as a unique time series being a base of characterization of severe geomagnetic storms. We showed that during every severe geomagnetic storm, the Katz fractal dimension of *E* grows rapidly, exactly at the same time when the value of the geoelectric field is increasing. When we calculated the Katz fractal characteristics of geomagnetic storms from the initial geomagnetic field components, $B_{X},B_{Y}$, we observed in general similar temporal changes as those that occurred for *E*, but with slightly higher values for the magnetic field case. A comparison of the results for modelled (*E*) and real (*B*) data suggests that the fractal and complex nature of the initial time series for the magnetic field is generally preserved, from the point of view of Katz’s methodology, during the transition from *B* to *E*. It has to be underlined that real observations of the geoelectric field are rare, as stated by, e.g., Beggan et al. (2021) [58]: “The paucity of widespread measurements of the geoelectric field elsewhere means it must instead be modelled”.

Summarizing, one can see that the calculated Katz fractal dimensions show a highly changeable level of complexity for each severe geomagnetic storm. Nevertheless, the Katz fractal dimension underestimates values for the quiet intervals; thus, it can be treated more as a storm classifier rather than a fractal dimension real value estimator. On one hand, the Katz fractal dimension appears to be far less sensitive to small changes, and on the other hand, the Higuchi method can be strongly unstable for perturbed time series; this may result in the Katz algorithm being more effective in detecting sharp signal changes, which are characteristic of geomagnetic storms, than the smaller, slower, and more gradual changes during quiet periods. We are convinced about the use of the Katz fractal dimension as a fast, useful indicator of distinct states of the geoelectric field. On the other hand, we underline that other methods should still be considered for a more appropriate characterization of the fractal structure of the analysed data. It is worth adding that the Katz fd adopted in the frame of our article was derived from a simple operation directly on the signal and not on any phase space. The consideration of geoelectric field data from dynamical system perspective states [21] is an idea worthy of further, systematic analysis.

Therefore, we plan to continue our analysis based on other approaches to the fractal dimension properties, first for the extreme G5 storms and later for the G3-strong storms. Moreover, we plan to consider more parameters in an attempt to study their relation with the fractal dimension obtained for the geoelectric field. This will allow to better characterize and classify individual storms. Furthermore, we plan to extend our analysis on the stations located at different latitudes.

In addition, we are aware that the 1-D layered Earth model has some limitations. In particular, two-dimensional and three-dimensional Earth conductivity structure can introduce some features not seen with 1-D models [85,86]. Therefore, in our future studies we plan to extend our work to other Earth modelling assumptions. 

## Data Availability

http://rtbel.igf.edu.pl/, https://www.gfz-potsdam.de/en/kp-index/ (accessed on 29 June 2021).
